# Supplementary material for: Highly Correlated Recurrence Prognosis in Patients with Metastatic Colorectal Cancer by Synergistic Consideration of Circulating Tumor Cells/Microemboli and Tumor Markers CEA/CA19-9
Source: Cells. 2021 May 10;10(5):1149. doi: 10.3390/cells10051149 (PMC8151024; doi:10.3390/cells10051149)
Supplement: Supplementary file 1 [file cells-10-01149-s001.zip › cells-1008242-supplementary.pdf]

Highly Correlated Recurrence Prognosis in Patients with Metastatic Colorectal Cancer by Synergistic Consideration of Circulating Tumor Cells/Microemboli and Tumor Markers

Hsueh-Yao Chu [1,9,†], Chih-Yung Yang[2,3,4,†], Ping-Hao Yeh[1], Chun-Jieh Hsu [1], Lu-Wei Chang [1], Wei-Jen Chan [1], Chien-Ping Lin [5], You-You Lyu [5], Wei-Cheng Wu [1,6], Chun-Wei Lee [1], Jen-Kuei Wu [1,7], Jeng-Kai Jiang [8,*] and Fan-Gang Tseng [1,6,10,11,*]

Recovering testing of rare cells was carried out for automatic SACA imaging system. HCT116 cells were diluted in white blood cells at different ratio from 1:10,000 to 1:1,000,000. HCT116 cells were stained by Hoechst 33258 positive/EpCAM-FITC positive/CD45-PECy-7 negative, respectively, and then dispensed into SACA chip. At 1:1,000,000 dilution., roughly 11% HCT116 cells were lost during preparation, and other single target HCT116 cells were clearly identified by the automatic SACA imaging system with an 89% recovery rate.

HCT-116 : WBC	Mixed HCT116 cells number	Detected HCT116 cells number	Average deviation value
1:10,000	32.8 ± 1.9	26	8%
		29	
		27	
1:100,000	31 ± 1.5	30	10%
		25	
		24	
1:1,000,000	35 ± 1.9	31	11%
		26	
		31	

Table 1. SACA image detecting system spike-in recovering rate.

CTC clusters were detected in PB of 14 CRC patients, (mean ± SEM, 1.07 ± 0.27) and MVB (mean ± SEM, 1.17 ± 0.51) of 16 CRC patients (Figure S1). The difference of CTC clusters numbers between PB and MVB was not statistically significant.

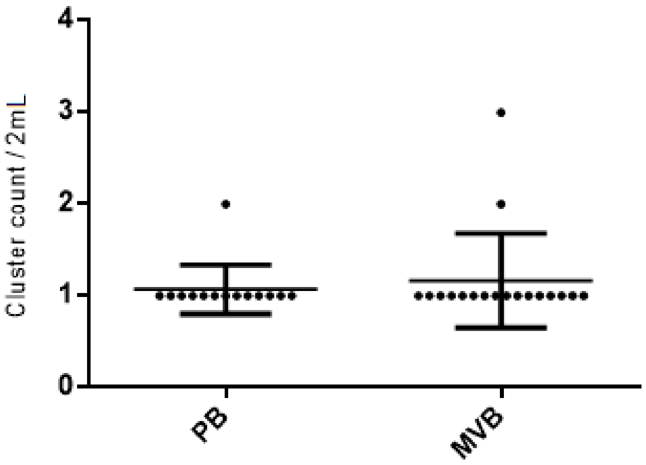

Figure S1. CTC clusters number of CRC patient in this research.

https://drive.google.com/drive/u/1/folders/1o8iNmdBTN_1___-DGO9iM9y21_tF2eFlV

Video S1. The image system protocol.
